# Chylous Ascites: A Rare Chronic Case

**DOI:** 10.7759/cureus.116384

**Published:** 2026-09-16

**Authors:** Regla M Silveira Rojas, Upasana Kapur, Aaditya Suryadevara, Sai K Avula

**Affiliations:** 1 Internal Medicine, Alabama College of Osteopathic Medicine, Dothan, USA; 2 Internal Medicine, Dothan Medical Associates, PC, Dothan, USA; 3 Faculty of Arts & Science, University of Toronto, Toronto, CAN; 4 Internal Medicine, Southeast Health Medical Center, Dothan, USA

**Keywords:** abdominal paracentesis, chylous ascites, cirrhosis, hypoalbuminemia, lymphedema

## Abstract

Chylous ascites (CA) is an uncommon form of ascites resulting from disruption or obstruction of the lymphatic system. Although cirrhosis and malignancy account for many cases in Western countries, management can be particularly challenging when the underlying etiology remains uncertain. We present the case of a 57-year-old man with chronic CA, longstanding unilateral right-sided lymphedema reportedly present since birth, and a prolonged clinical course spanning several years. Despite conservative management with diuretics, dietary modification, and repeated therapeutic paracenteses, the patient experienced recurrent symptomatic ascites and nutritional compromise. Ascitic fluid triglyceride testing supported the diagnosis of CA, and octreotide therapy was initiated. Although the patient's longstanding unilateral lymphedema raised concern for an underlying lymphatic abnormality, subsequent lymphangiography did not demonstrate a definitive lymphatic leak. A diagnosis of cirrhosis was also reported during a hospitalization abroad but was not confirmed by subsequent liver biopsy. The patient continues to require multidisciplinary management, including nutritional support, gastroenterology follow-up, and pharmacologic therapy, with a decrease in the frequency of therapeutic paracenteses. This case illustrates the diagnostic and therapeutic challenges of chronic CA when a definitive underlying etiology cannot be established and emphasizes the importance of comprehensive ascitic fluid analysis, nutritional optimization, and multidisciplinary evaluation in patients with persistent or refractory disease.

## Introduction

Chylous ascites (CA) is defined as the accumulation of chyle within the peritoneal cavity. Chyle is a milky, white, lipid-rich fluid containing proteins and lymphocytes. The underlying etiologies of CA include traumatic, congenital, infectious, neoplastic, postoperative, cirrhotic, and cardiogenic causes. These etiologies can be categorized as traumatic or nontraumatic and, from a pathophysiologic view, as portal or nonportal [1]. Portal causes are related to increased lymphatic pressure, while nonportal causes result from other underlying conditions. Approximately two-thirds of CA cases in Western countries are associated with abdominal malignancy and cirrhosis, whereas infectious etiologies, including tuberculosis and lymphatic filariasis, account for a substantial proportion of cases in developing countries [2].

The diagnosis of CA is based on the characteristics and analysis of the ascitic fluid obtained by paracentesis. Ascitic fluid triglyceride concentration is commonly used to support the diagnosis, although reported diagnostic thresholds vary among published studies. A triglyceride level greater than 200 mg/dL has traditionally been used as a diagnostic threshold [1,2]. However, subsequent studies have proposed lower thresholds, including 187 mg/dL as an optimal cutoff in one diagnostic study and ≥81 mg/dL in a study of patients with portal hypertension [3,4]. Lymphangiography can be both diagnostic and therapeutic by identifying sites of lymphatic leakage and allowing targeted embolization in selected cases [5].

Management focuses on treating the underlying disease process and careful attention to the patient’s nutritional status. A high-protein, low-fat diet with medium-chain triglyceride supplementation is important [2,6]. Patients usually require therapeutic paracentesis to manage symptoms. Pharmacologic therapies, including orlistat, somatostatin, octreotide, and etilefrine, have been described in the management of CA [2]. Surgical interventions in the form of transjugular intrahepatic portosystemic shunt (TIPS), peritoneal shunt, angiography with embolization of a leaking vessel, and laparotomy are options for cases refractory to medical management [2].

CA is a rare condition, with a reported incidence of approximately one in 20,000 admissions at a university-based hospital over 20 years [7]. More recently, a five-year retrospective study at a tertiary care hospital reported an estimated incidence of approximately 1 case per 10,000 hospitalizations [8]. Differences in study populations and methodology may account for the variation between these estimates. Chronic loss of chyle can result in malnutrition, lymphocyte depletion, and immunologic compromise, contributing to the morbidity associated with CA [1]. Reported mortality rates range from 40% to 70% and are largely influenced by the underlying etiology [1].

This case report highlights the diagnostic and therapeutic challenges of chronic CA concurrent with longstanding unilateral lymphedema and an uncertain underlying etiology. It emphasizes the importance of comprehensive ascitic fluid analysis, including triglyceride measurement, and multidisciplinary evaluation when ascites remains unexplained or is refractory to conventional management.

## Case presentation

After relocating from another state, a 57-year-old man presented to our primary care clinic with abdominal distention and left groin pain to establish care. His medical history was significant for type 2 diabetes mellitus and hypercholesterolemia. His medications included aspirin, atorvastatin, metformin, and glimepiride. His family history was notable for hypertension, diabetes mellitus, and cerebrovascular accident on his paternal side. He denied current or prior alcohol or tobacco use and followed a vegetarian diet. On physical examination, abdominal distention was noted without tenderness to palpation. Suprapubic fullness and scrotal edema were present. The right upper and lower extremities demonstrated 2+ pitting edema, which the patient reported had been present since birth. No hepatomegaly, splenomegaly, or lymphadenopathy was appreciated. During the initial visit, a gastroenterology referral was placed, and the patient was started on furosemide 40 mg orally as needed and omeprazole 40 mg daily. Dietary and lifestyle modifications were also recommended. Laboratory evaluation included a hepatitis B panel, which was negative; a comprehensive metabolic panel, which demonstrated hyperglycemia (glucose 289 mg/dL; reference range, 74-106 mg/dL), hyponatremia (sodium 127 mmol/L; reference range, 135-146 mmol/L), hyperkalemia (potassium 5.7 mmol/L; reference range, 3.5-5.3 mmol/L), low total protein (3.6 g/dL; reference range, 6.3-8.2 g/dL), and hypoalbuminemia (1.7 g/dL; reference range, 3.5-5.0 g/dL); and an unremarkable lipid panel. Urinalysis and urine microalbumin-to-creatinine ratio were normal. The complete blood count was unremarkable, and hemoglobin A1c was elevated at 11.3%. The patient's hyperkalemia precluded the use of spironolactone as initial therapy. At the time, further evaluation of the extremity edema was deferred due to insurance issues. Prior medical records were not available.

During the initial gastroenterology evaluation for persistent ascites, the patient reported no prior history of colonoscopy. A urine protein-to-creatinine ratio and 24-hour urine protein collection were negative. These results suggested that the patient was not losing protein through the urine. Computed tomography of the abdomen and pelvis demonstrated moderate ascites; however, the original images were unavailable to the authors. Prothrombin time (PT) was 10.5 seconds, with an international normalized ratio (INR) of 0.9 and a partial thromboplastin time (PTT) of 30.8 seconds. Platelet count was within normal limits. These findings provided no laboratory evidence of impaired hepatic synthetic function. A chronic hepatitis panel was also negative.

Over the following months, the patient returned for routine primary care follow-up and was referred to interventional radiology for diagnostic and therapeutic paracentesis. Ascitic fluid was obtained for cell count, Gram stain, bacterial cultures, pH, albumin, lactate dehydrogenase, and total protein analysis; however, specific laboratory values could not be obtained from the available records and appeared to have been lost in the laboratory. A computed tomography scan of the chest, obtained for evaluation of shortness of breath, demonstrated a large volume of ascites in the upper abdomen (Figure 1) as well as moderate-to-severe coronary artery calcification per the radiology report. The patient subsequently underwent multiple therapeutic paracenteses.

**Figure 1:**
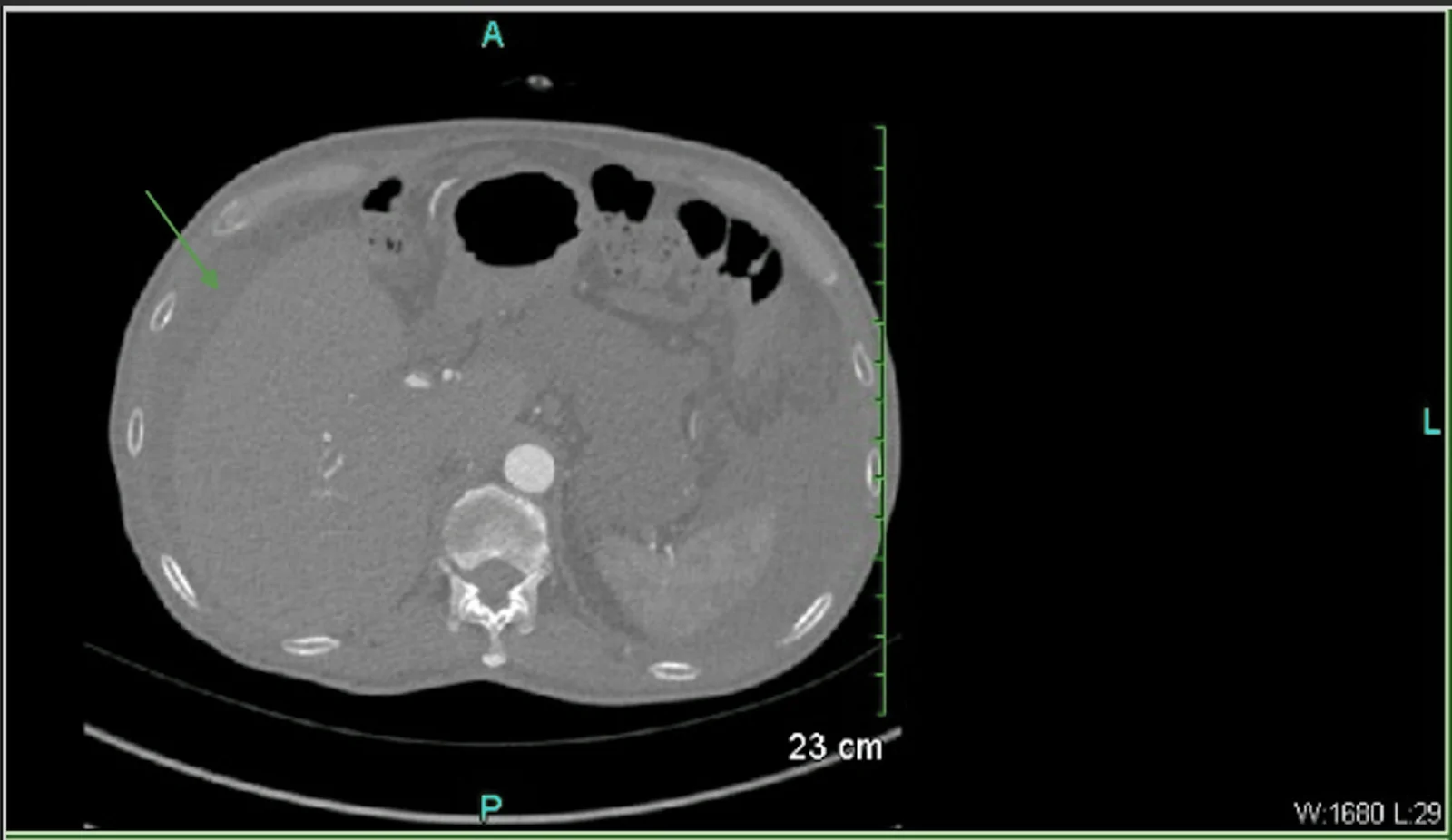
Large-volume ascites in the upper abdomen on computed tomography of the chest Representative axial computed tomography image of the chest demonstrating a large volume of ascites within the visualized upper abdomen (arrow).

During subsequent gastroenterology evaluation, the patient reported worsening suprapubic pain and persistent ascites. Laboratory evaluation demonstrated a serum albumin level of 1.7 g/dL and an ascitic fluid albumin level of less than 1.5 g/dL, yielding a serum-ascites albumin gradient (SAAG) greater than 1.1 g/dL. Ascitic fluid adenosine deaminase and mycobacterial PCR were negative, revealing no evidence of tuberculosis. No evidence of spontaneous bacterial peritonitis was identified either. Given the elevated SAAG, noncirrhotic portal hypertension versus a nonhepatic source of ascites was considered. Abdominal ultrasonography was recommended to evaluate for Budd-Chiari syndrome, and continued diuretic therapy was recommended while additional laboratory studies were pending. At follow-up with gastroenterology, FibroScan demonstrated no evidence of cirrhosis. The patient underwent additional paracentesis and was subsequently referred to a tertiary care center for further evaluation. The referral was initially declined because there was no evidence of cirrhosis.

Later that year, the patient developed clear drainage from the left lower extremity secondary to worsening edema. His furosemide dose was doubled temporarily for three days, and compression wrapping was recommended, resulting in improvement of edema and drainage. A cardiology referral was placed by gastroenterology. Cardiac evaluation was unrevealing, including a transthoracic echocardiogram that showed no evidence of heart failure. A graded exercise tolerance test demonstrated abnormal myocardial perfusion with evidence of an inferior scar/ischemia. No definitive source of the ascites was identified. Severe hypoalbuminemia secondary to suspected protein-losing enteropathy was considered by gastroenterology as a potential contributor to the patient's persistent ascites. Esophagogastroduodenoscopy and colonoscopy yielded negative biopsies in the duodenum and terminal ileum for lymphoma or enteropathy. Subsequent improvement of ascites led the patient to stop furosemide therapy.

Two years after initial presentation, the patient was hospitalized due to lymphadenitis with concern for compartment syndrome of the right upper extremity. Doppler ultrasonography and compartment pressure measurements were unremarkable; he was discharged after 24 hours. A similar episode occurred a year later, leading to a repeat ultrasound, which was negative. These events led to specialized lymphedema therapy consisting of compression bandaging of the right upper extremity, which led to significant improvement. His condition subsequently remained relatively stable, during which time he continued dietary and lifestyle interventions, including increased protein intake and exercise.

Three years after initial presentation, the patient traveled to India for several months, where he was hospitalized for worsening ascites, pleural effusions, hematemesis, and melena. According to the patient, he was told he had cirrhosis during this hospitalization abroad. Given these developments and the prolonged interval since his previous gastroenterology evaluation, the primary care clinic placed a new gastroenterology referral. Routine laboratory studies obtained during primary care follow-up continued to demonstrate hyponatremia and hypoalbuminemia. Hyperkalemia had resolved; spironolactone 100 mg and furosemide 40 mg orally daily were added to his diuretic regimen.

Three months later, the patient was admitted to the hospital for fluid overload, groin swelling, shortness of breath, and fatigue. Computed tomography of the abdomen demonstrated a cirrhotic-appearing liver with a patent portal vein and moderate abdominal ascites (Figure 2). Therapeutic paracentesis was performed, yielding 1.3 L of white, purulent-appearing ascitic fluid. Spontaneous bacterial peritonitis was not supported by an ascitic fluid neutrophil count <250 cells/mm³ and negative bacterial cultures. Up to this point, the appearance of the ascitic fluid was not consistently documented in the paracentesis reports. When described, the fluid was characterized as white, milky, or cloudy. The patient was subsequently observed and managed medically. His BMI was 18 kg/m² at the time, raising concern for nutritional compromise associated with his chronic disease despite efforts to increase protein intake.

**Figure 2:**
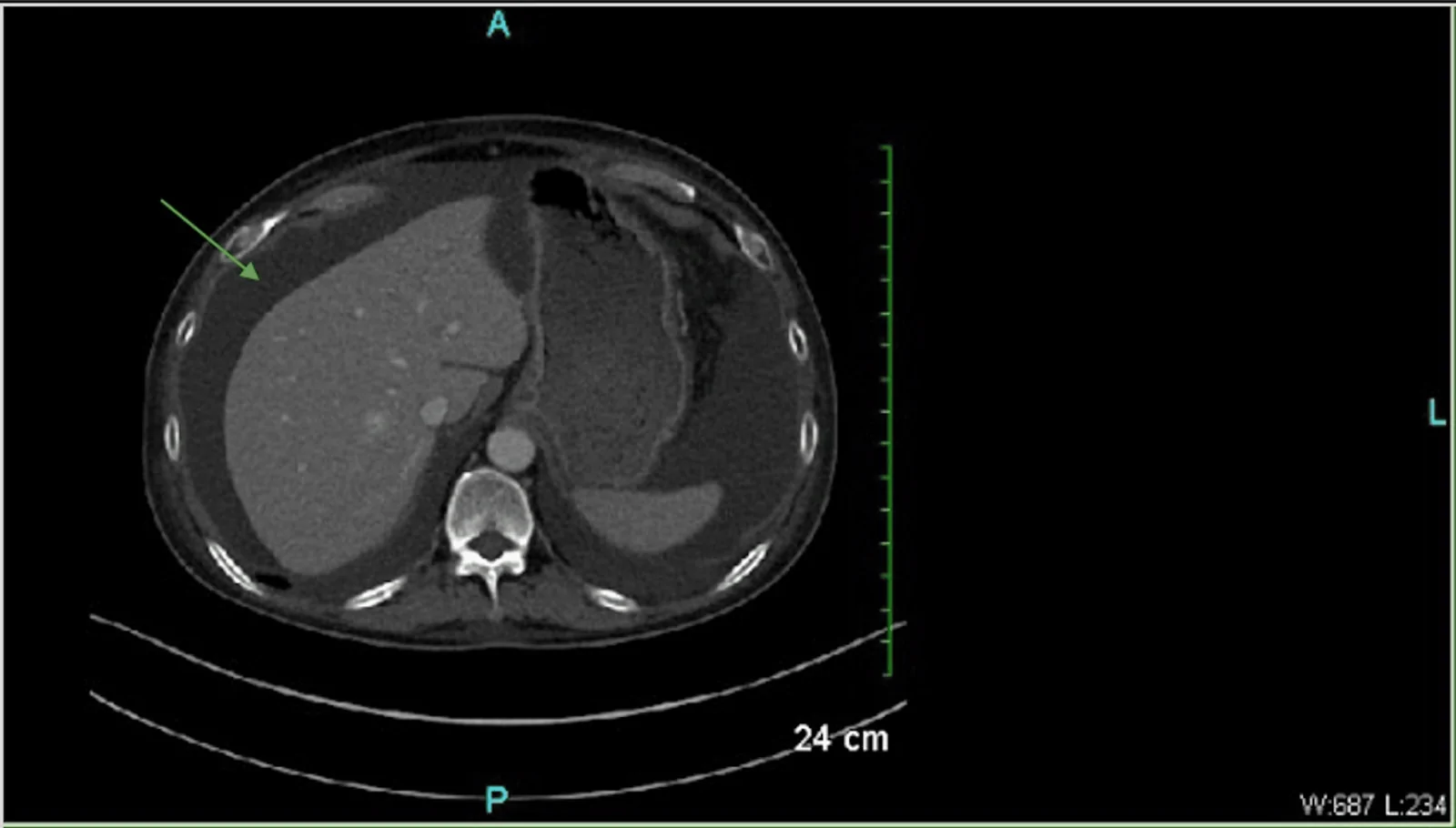
Computed tomography of the abdomen demonstrating moderate ascites Representative axial computed tomography image demonstrating moderate ascites surrounding the liver (arrow). The study also demonstrated cirrhotic changes of the liver.

During the gastroenterology evaluation for the cirrhosis diagnosed abroad, the previously described white, purulent-appearing ascitic fluid raised concern for CA and a possible malignant etiology. Endoscopic ultrasound with liver biopsy demonstrated minimal ascites and normal-appearing left hepatic lobe parenchyma; liver pathology was negative for cirrhosis. No esophageal varices were noted. Liver profile and hepatic function testing were unremarkable, and tuberculosis testing was negative once again. Flow cytometry showed no evidence of a hematologic malignancy. Paracentesis performed during this evaluation demonstrated an ascitic fluid triglyceride level of 126 mg/dL, white blood cell count of 98 cells/mm³, red blood cell count of 29 cells/mm³, and albumin level of 0.1 g/dL. Gram stain showed no organisms, and bacterial cultures demonstrated no growth. The gastroenterology team diagnosed CA based on the overall clinical presentation and ascitic fluid characteristics. The etiology had not been identified. Octreotide 100 mcg subcutaneously three times daily was initiated, with a recommendation to monitor ascitic fluid triglyceride levels during subsequent paracenteses.

During primary care follow-up, the patient reported that his referral to a tertiary care center had been approved and that he had undergone further evaluation. According to the patient, the tertiary care team suspected a lymphatic leak in the bilateral groin region, and a lymphangiogram was scheduled. Diuretic therapy was discontinued because the patient experienced fatigue and dizziness during paracentesis. Given his reported history of residence in India, a filarial antibody (IgG4) test was obtained and was negative (1.25; reference <2.50), making lymphatic filariasis a less likely cause. The lymphangiogram was subsequently performed and did not demonstrate a lymphatic leak.

At the most recent gastroenterology follow-up, the frequency of therapeutic paracentesis had decreased from every two weeks to every six weeks. The patient was transitioning from octreotide 100 mcg administered subcutaneously three times daily to lanreotide 60 mg administered subcutaneously every four weeks. Gastroenterology planned to contact the tertiary care center to coordinate further management. To date, no underlying etiology for the CA has been identified. Table 1 summarizes the major clinical events, diagnostic evaluations, and management over the course of the patient’s illness.

**Table 1:** Chronological timeline of major clinical events, evaluation, and management GI, gastrointestinal; IgG4, immunoglobulin G4; INR, international normalized ratio; PO, by mouth (per os); PT, prothrombin time; SAAG, serum-ascites albumin gradient; SBP, spontaneous bacterial peritonitis; SQ, subcutaneous; TB, tuberculosis; TID, three times daily; US, United States

Time	Major Clinical Event	Key Evaluation / Management / Outcome
Initial presentation	Ascites, abdominal distention, groin pain, and longstanding right-sided limb edema.	Severe hypoalbuminemia without urinary protein loss; furosemide 40 mg PO started and GI referral placed; minimal symptomatic relief.
Early course	Persistent/recurrent ascites requiring paracenteses.	FibroScan and PT/INR showed no evidence of cirrhosis or impaired hepatic synthetic function. SAAG >1.1; TB and SBP evaluations negative.
Years 1-3	Progressive extremity edema with recurrent right upper-extremity swelling and episodes requiring hospitalization.	Cardiac and GI evaluations were unrevealing. Lymphedema therapy with compression bandaging improved significantly. Patient stopped furosemide therapy.
Year 3	Hospitalized in India for worsening ascites, pleural effusions, hematemesis, and melena; reported diagnosis of cirrhosis.	Persistent hypoalbuminemia and hyponatremia. Diuretics restarted after return to the US.
3 months later	Hospitalized for fluid overload, groin swelling, dyspnea, and fatigue; white/milky/cloudy ascitic fluid noted.	CT showed a cirrhotic-appearing liver, but liver biopsy showed no cirrhosis. Ascitic fluid did not support SBP; triglycerides 126 mg/dL. Chylous ascites diagnosed and SQ octreotide 100 mcg TID.
Most recent follow-up	Lymphatic etiology investigated; cause remained unidentified.	Filaria IgG4 negative and lymphangiography showed no leak. Paracentesis interval prolonged from every 2 to every 6 weeks; transitioning to lanreotide.

## Discussion

The diagnosis of CA in this patient required consideration of the variability in reported thresholds for ascitic fluid triglyceride levels. His ascitic fluid triglyceride level was 126 mg/dL, which was below the traditionally cited threshold of 200 mg/dL. However, published studies have demonstrated variability in the triglyceride concentration used to support the diagnosis. One study identified 187 mg/dL as the optimal cutoff for peritoneal fluid, while another study in patients with portal hypertension reported a threshold of ≥81 mg/dL with high sensitivity and specificity when chylomicron detection was used as the reference standard [3,4]. Ascitic fluid was also described as milky white, a characteristic finding associated with CA. Whether chylomicron testing was performed during the patient's evaluation could not be determined from the available records. This is a limitation. The diagnosis of CA was made by the gastroenterology team based on the overall clinical presentation and ascitic fluid characteristics, including its milky-white appearance and triglyceride concentration. Given the variability in reported diagnostic thresholds and the clinical context, the diagnosis was considered consistent with CA despite the triglyceride level being below 200 mg/dL.

Somatostatin analogs, including octreotide, have been used as adjunctive therapy for CA when conservative measures are insufficient, although the available evidence is largely limited to case reports and small case series [9]. In this patient, octreotide 100 mcg administered subcutaneously three times daily was initiated after persistent ascites despite conservative management. At the most recent follow-up, the patient was transitioning from octreotide to lanreotide, a long-acting somatostatin analog administered every four weeks. This change likely aimed to reduce the treatment burden of multiple daily injections rather than provide a demonstrated efficacy advantage. Although lanreotide and octreotide share similar somatostatin receptor activity, evidence specifically supporting lanreotide for CA remains limited. Therefore, the use of lanreotide in this case applies the therapeutic effects of somatostatin analogs, with the potential practical advantage of less frequent administration.

Several factors complicated this patient's clinical course, including longstanding unilateral lymphedema, recurrent CA, uncertainty about the underlying etiology, and nutritional difficulties. Of particular interest was the patient's reported lifelong unilateral lymphedema involving the right upper and lower extremities, which initially raised concern for an underlying lymphatic abnormality. Prior studies have demonstrated an association between lymphedema and disorders of lymphatic flow. In a case series of 45 patients with CA, 35 had primary CA associated with lymphatic abnormalities, while additional lymphatic abnormalities were identified in 36 patients; lymphedema of the leg was the most common, occurring in 26 patients [10]. In another series of 35 patients with primary chylous disorders, reflux of chyle into the pelvic or lower-extremity lymphatics was associated with lymphedema in 14 of 16 patients and cutaneous lymphatic fistulae in 11 patients [11].

In our patient, the longstanding unilateral lymphedema, groin and scrotal involvement, and episodes of lower-extremity lymphatic drainage raised clinical suspicion for an underlying lymphatic disorder. Given this persistent clinical suspicion, lymphangiography was pursued. In a study of 31 patients with refractory CA, lymphangiography identified a site of lymphatic leakage in 55% of patients, and lymphatic embolization resulted in resolution of CA in 82% [5]. In contrast, lymphangiography in our patient did not demonstrate a definitive lymphatic leak, limiting the availability of targeted interventional treatment. Although the patient's clinical findings raised suspicion for an underlying lymphatic abnormality, a definitive association between the patient's longstanding lymphedema and CA could not be established. Furthermore, there was no evidence to confirm that the patient's CA was attributable to a congenital lymphatic abnormality.

The patient's hepatic evaluation further complicated the determination of the underlying etiology. Although he was reportedly diagnosed with cirrhosis during a hospitalization abroad, and in the US a CT of the abdomen suggested cirrhotic changes of the liver, subsequent FibroScan and liver biopsy did not demonstrate cirrhosis. Liver profile and hepatic function testing were also unremarkable. This evaluation was considered important because portal hypertension can substantially increase hepatic and gastrointestinal lymph production and contribute to the development of CA [1]. The absence of histopathologic evidence of cirrhosis did not support chronic liver disease.

One important complication of chronic CA is persistent loss of chyle, which can result in substantial losses of protein, lymphocytes, immunoglobulins, and other nutrients [6]. These losses may contribute to malnutrition and impaired immune function, making nutritional support an important component of management [6]. In our patient, nutritional compromise became evident through hypoalbuminemia, a BMI of 18 kg/m², the need for ongoing dietary counseling, and protein supplementation. A systematic review of nutritional management in patients with chyle leakage found that 72% of 550 patients achieved resolution without surgical intervention, although the available evidence did not establish the superiority of a specific dietary strategy [12]. The patient's vegetarian dietary preferences further complicated maintaining adequate nutritional intake. This case emphasizes that managing chronic CA extends beyond controlling fluid accumulation and requires ongoing attention to nutritional status, particularly when conservative treatment is prolonged.

A strength of this case is its longitudinal follow-up, which provides insight into the challenges of managing CA over several years. Rather than representing a single hospitalization or isolated presentation, this case demonstrates the complexity of persistent CA despite repeated diagnostic evaluations and therapeutic interventions. The patient's course required coordination among primary care, gastroenterology, cardiology, nutrition, lymphedema therapy, and tertiary care specialists. Importantly, extensive evaluation did not establish a definitive underlying cause despite consideration of hepatic, cardiac, infectious, malignant, and lymphatic etiologies.

Several clinical lessons can be drawn from this case. First, comprehensive analysis of ascitic fluid during the initial paracentesis is important, along with consistent documentation of the fluid's appearance during subsequent procedures. Second, close communication and collaboration among specialties are essential and should extend beyond the exchange of information through the electronic medical record (EMR). Finally, patients may not always present within established diagnostic thresholds, and clinical management should be guided by the overall clinical presentation rather than a single laboratory value, particularly when a definitive etiology remains uncertain. In our patient, the diagnosis of CA evolved over several years, while the underlying etiology remains uncertain despite extensive evaluation.

## Conclusions

CA is a rare condition that may require prolonged conservative management and, in refractory cases, more invasive diagnostic and therapeutic interventions. This case highlights the challenges of managing chronic CA in a patient with longstanding unilateral lymphedema, nutritional compromise, and no definitive underlying etiology despite extensive evaluation. Management required repeated therapeutic paracenteses, dietary and nutritional support, pharmacologic therapy, and multidisciplinary coordination. This case emphasizes the importance of maintaining a broad differential diagnosis, obtaining comprehensive ascitic fluid analysis, including triglyceride measurement, and pursuing timely specialized evaluation when ascites remains recurrent or refractory to conventional management.
